# Simulated Atmospheric N Deposition Alters Fungal Community Composition and Suppresses Ligninolytic Gene Expression in a Northern Hardwood Forest

**DOI:** 10.1371/journal.pone.0020421

**Published:** 2011-06-20

**Authors:** Ivan P. Edwards, Donald R. Zak, Harald Kellner, Sarah D. Eisenlord, Kurt S. Pregitzer

**Affiliations:** 1 School of Natural Resources and Environment, University of Michigan, Ann Arbor, Michigan, United States of America; 2 Department of Ecology and Evolutionary Biology, University of Michigan, Ann Arbor, Michigan, United States of America; 3 College of Natural Resources, University of Idaho, Moscow, Idaho, United States of America; Research Institute for Children and the Louisiana State University Health Sciences Center, United States of America

## Abstract

High levels of atmospheric nitrogen (N) deposition may result in greater terrestrial carbon (C) storage. In a northern hardwood ecosystem, exposure to over a decade of simulated N deposition increased C storage in soil by slowing litter decay rates, rather than increasing detrital inputs. To understand the mechanisms underlying this response, we focused on the saprotrophic fungal community residing in the forest floor and employed molecular genetic approaches to determine if the slower decomposition rates resulted from down-regulation of the transcription of key lignocellulolytic genes, by a change in fungal community composition, or by a combination of the two mechanisms. Our results indicate that across four *Acer*-dominated forest stands spanning a 500-km transect, community-scale expression of the cellulolytic gene *cbh*I under elevated N deposition did not differ significantly from that under ambient levels of N deposition. In contrast, expression of the ligninolytic gene *lcc* was significantly down-regulated by a factor of 2–4 fold relative to its expression under ambient N deposition. Fungal community composition was examined at the most southerly of the four sites, in which consistently lower levels of *cbh*I and *lcc* gene expression were observed over a two-year period. We recovered 19 basidiomycete and 28 ascomycete rDNA 28S operational taxonomic units; *Athelia*, *Sistotrema*, *Ceratobasidium* and *Ceratosebacina* taxa dominated the basidiomycete assemblage, and Leotiomycetes dominated the ascomycetes. Simulated N deposition increased the proportion of basidiomycete sequences recovered from forest floor, whereas the proportion of ascomycetes in the community was significantly lower under elevated N deposition. Our results suggest that chronic atmospheric N deposition may lower decomposition rates through a combination of reduced expression of ligninolytic genes such as *lcc*, and compositional changes in the fungal community.

## Introduction

Elevated levels of atmospheric nitrogen (N) deposition, resulting from anthropogenic activity, are a global phenomenon and lead to increased N availability in terrestrial ecosystems, such as temperate forests in which plant growth is generally N limited [1], [2]. Higher N availability may increase carbon (C) storage in these ecosystems by stimulating primary productivity [3], [4], [5] and by increasing soil C sequestration [4], [6], [7]. Because of its size and temporal stability, understanding the long-term impact of N deposition on the soil C storage is particularly important, because it is a major pool in the global C cycle. Nitrogen deposition could increase soil C sequestration by increasing aboveground litter production, increasing root litter production, or reducing microbial activity. We have recently demonstrated that simulated atmospheric N deposition, at a rate expected by 2050 across portions of the Northern Hemisphere [1], has increased net primary productivity and soil C sequestration (∼100 g C m^−2^ y^−1^ from 1994 to 2004) in a widely distributed temperate deciduous forest ecosystem [8]. In this ecosystem, increased soil C sequestration has occurred despite no significant increase in above- or belowground litter production, but concomitant with declines in microbial lignocellulolytic extracellular enzyme activities and with the accumulation of organic matter in the forest floor [8], [9], [10]. Because N deposition stimulation of C sequestration in this ecosystem appears to be mediated through the saprotrophic microbial community, in this study we employed molecular genetic approaches to examine the mechanisms underlying this response.

Litter decomposition is primarily a biochemical process, and litter biochemistry, especially the relative proportions of cellulose and lignin, strongly affects decomposition rates and also the magnitude and direction of the response of decomposition rate to increased N availability [11]. Cellulose, which is a glucose polymer, is the main constituent of plant cell walls and is broken down through the action of cellulolytic enzymes (cellulases; i.e. cellobiohydrolases, endo-glucanases, and β-glucosidases). Lignins are phenolic polymers integral to plant secondary cell walls, and they are mineralized by a range of lignolytic enzymes, including lignin-peroxidases, manganese-peroxidases, and laccases [12]. In the forest floor, these lignocellulolytic enzymes are of predominantly fungal origin and are secreted by a phylogenetically wide range of species [13], [14], [15]. The saprotrophic fungal community is species rich and highly variable in space and time, reflecting environmental variation [16] as well as life-strategy differences between species and the continuously changing nature of the substrate as decomposition progresses [17]. Higher levels of N availability have the potential to affect the composition of the fungal community [18], and in doing so might alter lignocellulolytic enzyme production. For example, a declining proportion of basidiomycete species in the community has been postulated as a cause of lower decomposition rates, because saprotrophic basidiomycetes are often considered the primary agents of lignin decomposition [18], [19], [20].

However, higher N availability may also directly affect the transcription of the functional genes that encode for lignocellulolytic enzymes [21], [22] and in doing so affect decomposition without necessarily eliciting a change in community composition. The expression of cellulolytic genes is induced by the presence of cellulose, and repressed by elevated concentrations of simple sugars [21], [23], [24]. Higher N availability has been associated with increased cellulolytic gene transcription [21] and with higher levels of cellulase activity [11], [15]. As decomposition progresses, the fraction of cellulose bound in recalcitrant complexes with lignin and other polyphenols increases, and further mass loss becomes increasingly controlled by the rate at which lignin is metabolized [13]. Ligninolytic activity has been connected to nutrient depletion [25], [26], and the expression of lignin- and Mn-peroxidases may be repressed by higher N availability [27]. Laccase expression displays a more variable response to increased N availability, with repressed expression occurring in some fungal species and stimulated expression in others [28], [29]. The effect of N availability on the transcription of ligninolytic and cellulolytic genes has only been considered for a small number of species *in vitro*, and, to our knowledge, has not been examined under field conditions. In this study, we sampled forest floor from sugar-maple (*Acer saccharum* Marsh.) dominated forest stands in which chronic simulated N deposition (30 kg NO_3_- -N ha^−1^ yr^−1^since 1994) has been associated with lower levels of lignocellulolytic enzyme activity and increased levels of C sequestration. We tested three alternate hypotheses: 1) simulated N deposition represses lignocellulolytic gene transcription, 2) simulated N deposition alters fungal community composition, 3) declines in decay rates result from a combination of both

## Results

### Lignocellulolytic gene expression

**Figure 1:**
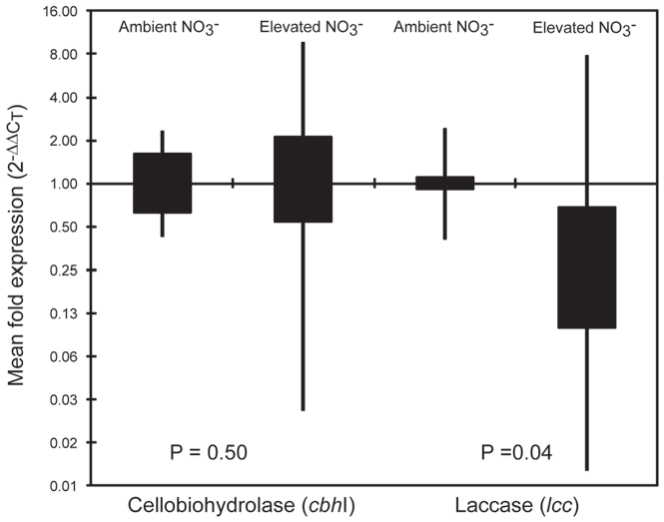
Relative expression of fungal cbhI and lcc genes in the forest floor of northern hardwood forests under ambient or elevated levels of N deposition. Boxes define the interquartile range, whiskers mark the minimum and maximum observations; relative expression (Y-axis) is log-scaled. *P*-values are significance of the mean difference in relative expression under ambient and elevated N deposition (Mann-Whitney U, n = 21).

The expression of the lignocellulolytic genes *cbh*I and *lcc* in the forest floor of experimental plots receiving elevated levels of N was determined relative to that of forest floor in plots receiving ambient levels of N deposition at one sugar-maple dominated site (Site D, Table 1) in 2007 and 2009, and also at an additional three sugar-maple dominated sites (A – C, Table 1) in 2009. Cellobiohydrolase and laccase enzyme activities were determined in parallel. Beta-tubulin, *cbh*I, and *lcc* genes were successfully amplified from 27 of 30 cDNA (6 in 2007 and 21 in 2009). The expression of the cellobiohydrolase *cbh*I gene relative to β-tubulin (ΔCT *^cbh^* ^I−*β*tub^) varied between sites and showed no clear relationship to N deposition (Table 2). Normalized relative *cbh*I expression (2^−ΔΔCT^) ranged from 0.06- to 6.85-fold across the four sites in 2009, and was not significantly affected by N deposition (Mann-Whitney U  = 46, n_ambient_  = 9, n_simulated N deposition_  = 11, *P*_two-tailed_  = 0.50, Fig. 1). At Site D, despite a high degree of spatial variation (0.23-fold to 6.25-fold in 2007; 0.09-fold to 4.54-fold in 2009) *cbh*I expression levels were significantly lower under simulated N deposition in the pooled two-year dataset (Mann-Whitney U  = 35, n_ambient_  = n_simulated N deposition_  = 6, *P*_two-tailed_  = 0.01). Laccase gene expression also showed considerable variation, although mean ΔCT values were generally lower under simulated N deposition (Table 2). Normalized *lcc* gene expression (2^−ΔΔCT^) ranged from 0.02-fold to 11.5-fold across the four forest sites in 2009, and tended to be lower under simulated N deposition (Fig. 1). Despite the high variability in mean fold expression, non-parametric analysis indicated that *lcc* gene expression was significantly lower under simulated N deposition (Mann-Whitney U  = 21, n_ambient_  = 9, n_simulated N deposition_  = 11, *P*_two-tailed_  = 0.04), providing evidence supporting one of our hypotheses. Laccase gene expression was also significantly lower under simulated N deposition at Site D in the pooled two-year dataset (Mann-Whitney U  = 27, n_ambient_  = 6; n_simulated N deposition_  = 5, *P*_two-tailed_  = 0.04).

**Table 1:** Site, climatic, overstory and ambient nitrogen deposition rates of four sugar maple stands receiving experimental NO_3_^-^ additions.

Forest characteristics	Site A	Site B	Site C	Site D
Latitude, N	46°52′	45°33′	44°23′	43°40′
Longitude, W	88°53′	84°51′	85°50′	86°09′
Mean annual precipitation, mm	821	828	856	793
Mean annual temperature, °C	4.8	6.1	6.9	7.6
Wet plus dry total N deposition kg ha^−1^ yr^−1^	6.8	9.1	11.7	11.8
Total N deposited, 1994–2009 kg ha^−1^ yr^−1^	590	625	667	670
Overstory age, 2009	102	96	97	101
Sugar maple % of overstory biomass	91	86	79	71

**Table 2:** Cellobiohydrolase and laccase enzyme activities and gene expression levels under ambient and elevated NO_3_^−^ deposition in four northern hardwood forests.

	Cellobiohydrolase		Laccase	
Site/Treatment	Enzyme activity(mean ± SD)nmol h^−1^ g^−1^	*cbh*I expressionΔCT ^(*cbh*I –βtub)^(mean ± SD)	Enzyme activity(mean ± SD)nmol h^−1^ g^-1^	*lcc* expressionΔCT ^(*lcc* – βtub)^(mean ± SD)
A/ambientA/simulated N	1334±664810±194	1.70±1.560.47±0.36	79±6832±13	3.90±1.825.36±0.31
B/ambientB/simulated N	992±175524±159	1.46±0.233.52±2.81	290±20645±22	7.81±0.039.75±4.59
C/ambientC/simulated N	839±1741104±194	4.14±0.953.33±2.20	161±13724±148	5.45±0.205.50±1.94
D/ambient _(2009)_D/simulated N _(2009)_ D/ambient _(2007)_D/simulated N _(2007)_	1003±71836±205427±42293±125	3.41±1.235.99±2.683.98±0.716.71±0.65	232±11576±1791±2577±11	2.36±0.977.36±3.541.30±0.691.46±0.80
P (α = 0.05) simulated N ≠ ambient N^1^	0.14	0.50	0.14	0.04

1 2009 data.

Cellobiohydrolase enzyme activity across the four sites in 2009 was on average 22% lower under simulated N deposition than under ambient N deposition (Table 2), although this was not statistically significant (Mann^−^Whitney U = 46, n_ambient_  =  n_simulated N deposition_  = 12, *P*_two-tailed_  = 0.14). Cellobiohydrolase activity was significantly different between years at Site D (mean (± SD) _2007_, 360 (±111) nmol h^−1^ g^−1^; mean (± SD) _2009_, 920 (± 165) nmol h^−1^ g^−1^; *P* = 0.01). Despite the temporal difference in magnitude, cellobiohydrolase activity tended to be lower under simulated N deposition than under the ambient condition (Table 2), and this was statistically significant (Mann-Whitney U = 32, n_ambient_  = n_simulated N deposition_  = 6, *P*_two-tailed_  = 0.03).

Laccase enzyme activity varied considerably across the four sites in 2009 (Table 2); it was lower under simulated N deposition than under ambient N deposition at three of the four sites, although overall N deposition had no significant effect (Mann-Whitney U = 46, n_ambient_ =  n_simulated N deposition_  = 12, *P*_two-tailed_  = 0.14). Laccase enzyme activity was higher in 2009 than in 2007 at Site D (Table 2), although this temporal difference was not significant (*t*-test, *P* = 0.19, *n* = 10). In each year, laccase activity tended to be lower under simulated N deposition than under ambient N deposition, but this was not statistically significant (Mann-Whitney U = 30, n_ambient_ =  n_simulated N deposition_  = 6, *P*_two-tailed_  = 0.06).

### Fungal Community Composition

**Figure 2:**
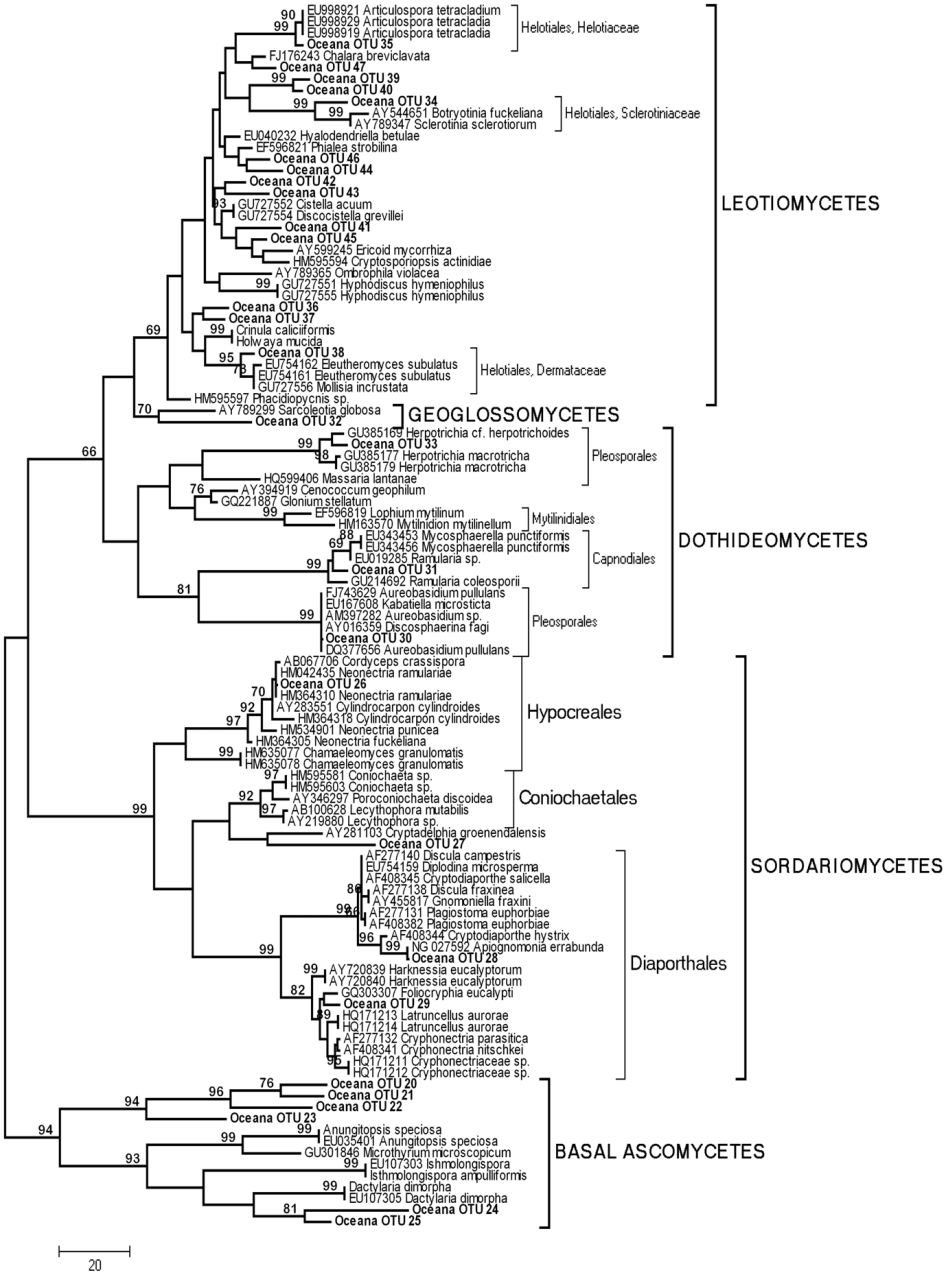
Phylogenetic relationships between 28 environmental Ascomycete sequences recovered from a maple-dominated hardwood site (Site D, “Oceana”) and 79 representative Ascomycete sequences recovered from GenBank. Tree represents the 50% consensus of 43 most parsimonious trees (tree length 1391) inferred from *ca*. 500 bp at the 5′ end of the nuclear large subunit. MP bootstrap values >65% are shown above nodes.

**Figure 3:**
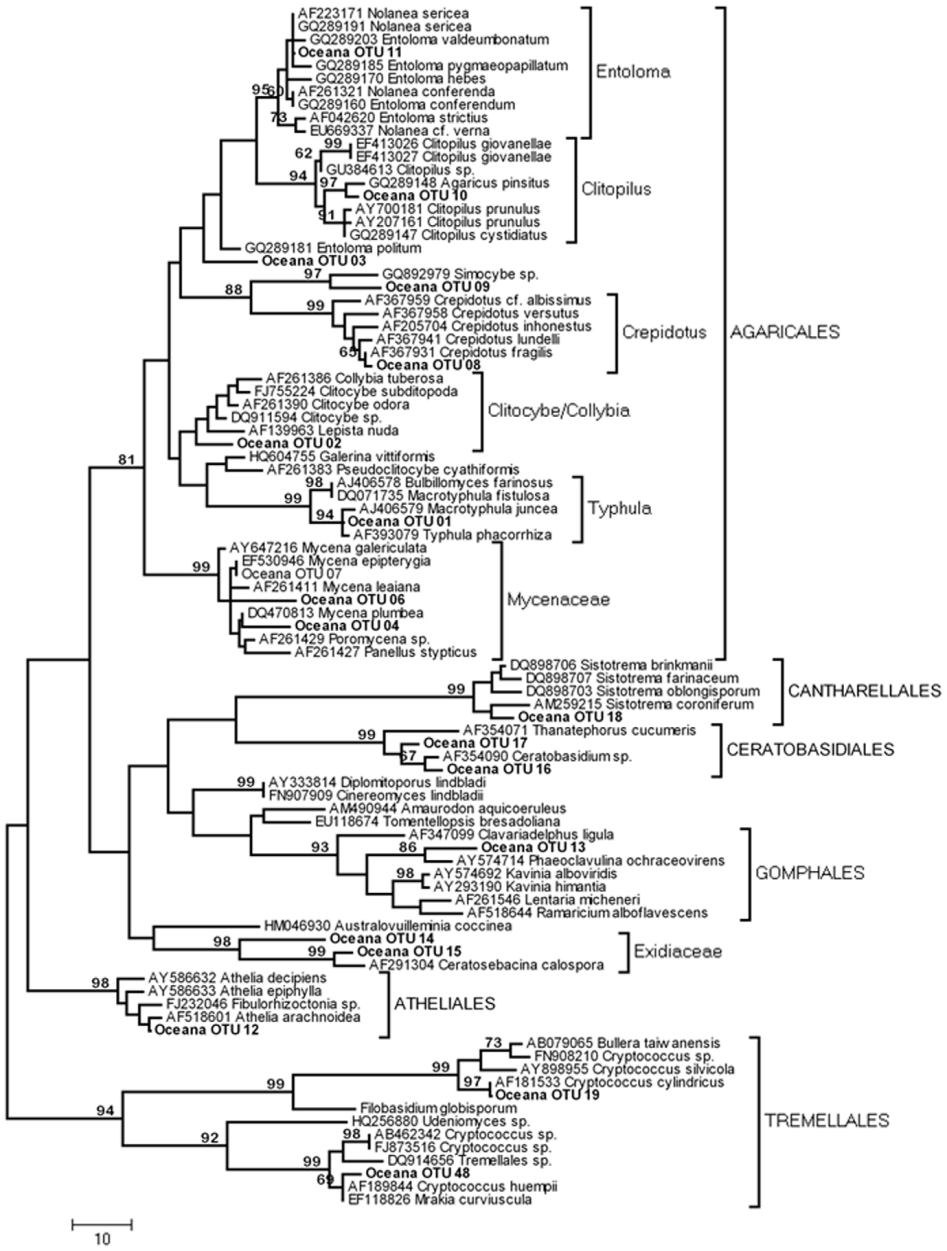
Phylogenetic relationships between 19 environmental Basidiomycete sequences recovered from a maple-dominated hardwood site (Site D, “Oceana”) and 73 representative Basidiomycete sequences recovered from GenBank. Tree represents the 50% consensus of 33 most parsimonious trees (tree length 885) inferred from *ca*. 500 bp at the 5′ end of the nuclear large subunit. MP bootstrap values >65% are shown above nodes.

The response of fungal community composition to chronic N deposition was examined at Site D. We obtained high quality sequence data from 209 of 288 rDNA 28S clones (72.5%), and 203 of these sequences were of fungal origin (97%). Phylogenetic analyses divided the fungal sequences into 47 Operational Taxonomic Units (OTUs). Ascomycotina dominated the OTUs (28 OTU *vs*. 19 Basidiomycota), yet they composed only 24% of the fungal sequences. This discrepancy was driven by the abundance of two basidiomycete OTUs (*Ceratobasidium* sp. and *Athelia* sp.) which were widespread (each recovered from five of six plots within the site) and whose combined abundance accounted for 46% of the fungal sequences. Phylogenetic analyses indicated that Pezizomycotina dominated the Ascomyctona, with Leotiomycetes, Dothideomycetes, Sordariomycetes and Geoglossomycetes taxa representing a combined 82% of the library (Fig. 2). Leotiomycetes represented 50% of ascomycete diversity (Fig. 2) and the three most widespread and abundant ascomycete OTUs recovered placed within this group. Within the Basidiomycota, species from at least seven orders within the Agaricomycotina were recovered in cDNA (Fig. 3). Agaricales dominated diversity with 10 of 19 OTUs (Fig 3), although the most widespread and abundant OTUs placed in the Ceratobasidiales, Atheliales, and Auriculariales. The proportion of ascomycete taxa in the community was significantly smaller under simulated N deposition (mean ambient, 63%; mean elevated NO_3_^−^, 37%; t-test, *P*_2-tailed_  = 0.02, n = 5).

Of the 47 fungal OTUs, only 12 (8 Basidiomycotina, 4 Ascomycotina) were recovered from more than one of the six field plots, and only 6 occurred under both ambient and simulated N deposition. We used Canonical Correspondence Analysis (CCA) to determine if the relative abundance of these 12 most widespread taxa was affected by N^−^ deposition, using plot levels of soil moisture content as a covariable. The principal CCA axis (constrained) accounted for 23% of the variability in species relative abundance; the second axis (unconstrained) for a further 37% (Fig. 3). There was no evidence that simulated N deposition at this site significantly affected the relative abundance of the more common fungal species (Monte Carlo *P*_principal axis_  = 0.60).

## Discussion

Litter decomposition is an enzymatically complex process mediated by a species-rich community of saprotrophic fungi [13], [30], and litter decomposition rates are known to be sensitive to N availability in the environment [7], [11], [19], [31]. Nitrogen availability is also known to affect the transcription of fungal genes that encode for the enzymes critical to the decomposition process [22], [25], [26], [27] and therefore altered rates of gene transcription under elevated N deposition may mechanistically underlie ecosystem level responses to this agent of global change. Also, because co-occurring fungal species differ in their rates, modes and potential for lignocellulolytic activity [14], [15], [32], altered community composition resulting from chronic elevated N deposition might also mechanistically underlie changes in litter decomposition rates and C sequestration. These mechanisms are not mutually incompatible, and, in this study, we examined levels of gene transcription across a series of northern hardwood forest stands receiving experimental N deposition for over a decade, and moreover examined community composition in a stand that displayed sustained levels of reduced lignocellulolytic gene expression under elevated N deposition. Our results indicate that although elevated N deposition can result in lower levels of both ligninolytic and cellulolytic gene transcription in the fungal communities of northern hardwood forest floor, ligninolytic gene transcription appears to respond in more negative manner.

### Functional Gene Transcriptional Response to Chronic N Deposition

We examined the community-scale transcriptional response of lignocellulolytic genes to simulated N deposition by focusing on the relative transcription levels of genes that encode for fungal cellobiohydrolase (*cbh*I) and laccase (*lcc*) enzymes. *The* c*bh*I gene is unique to fungi, broadly distributed between the Ascomycotina, Basidiomycotina and possibly Chytridiomycotina in forest soils [33], and encodes for an enzyme critical to cellulose breakdown. Expression of *cbh*I showed no consistent response to simulated N deposition across four study sites, although it was significantly lower under simulated N deposition at one of these sites over time; cellobiohydrolase enzyme activity likewise was not significantly affected by elevated N deposition. Nitrogen deposition has been associated with higher cellulolytic enzyme activities and higher initial rates of mass loss in cellulose-rich litters such as the predominantly sugar maple leaf litter of these sites [6]. We did not sample fresh litter in this study, but rather deliberately targeted the latter stage of litter decomposition of the Oe horizon. As such, our results suggest that during this latter stage of decomposition, higher N availability neither promotes nor represses expression of fungal cellobiohydrolase in the fungal community of this ecosystem.

In contrast to *cbh*I, community-scale expression of the *lcc* gene was significantly lower in the Oe horizon under simulated N deposition (Fig 1). Although laccase enzyme activity was significantly lower in previous studies [6], [9], the decline we document here was not statistically significant (Table 2). This apparent disconnect may reflect differences in the specificity of the gene-expression and enzyme activity assays, as tyrosinases or even bacterial laccase-like multicopper oxidases released during sample preparation [34] may also contribute to measured levels of phenol-oxidase activity [35]. Despite this, our gene-transcriptional and enzyme activity results exhibited strong congruence, with simulated N deposition predominantly associated with reduced expression of fungal *lcc* and lower overall laccase activity (Table 2). Although fungal laccases may play a role in morphogenesis and pathogen-host and fungal-fungal interactions, they are also an important component of the suite of oxido-reductive enzymes produced by fungi to break down lignin [35]. Lower levels of *lcc* expression in the Oe horizon under simulated N deposition may explain how the long-term trend towards lower phenol-oxidase activity observed in this ecosystem occurs despite no significant reduction in the average *lcc* gene copy number per gram of soil [9], [20]. As such, the transcriptional response of *lcc* to elevated levels of N availability may be an important mechanism underlying the slowing of decay and higher soil C sequestration [8].

### Fungal Community Response to Simulated N Deposition

**Figure 4:**
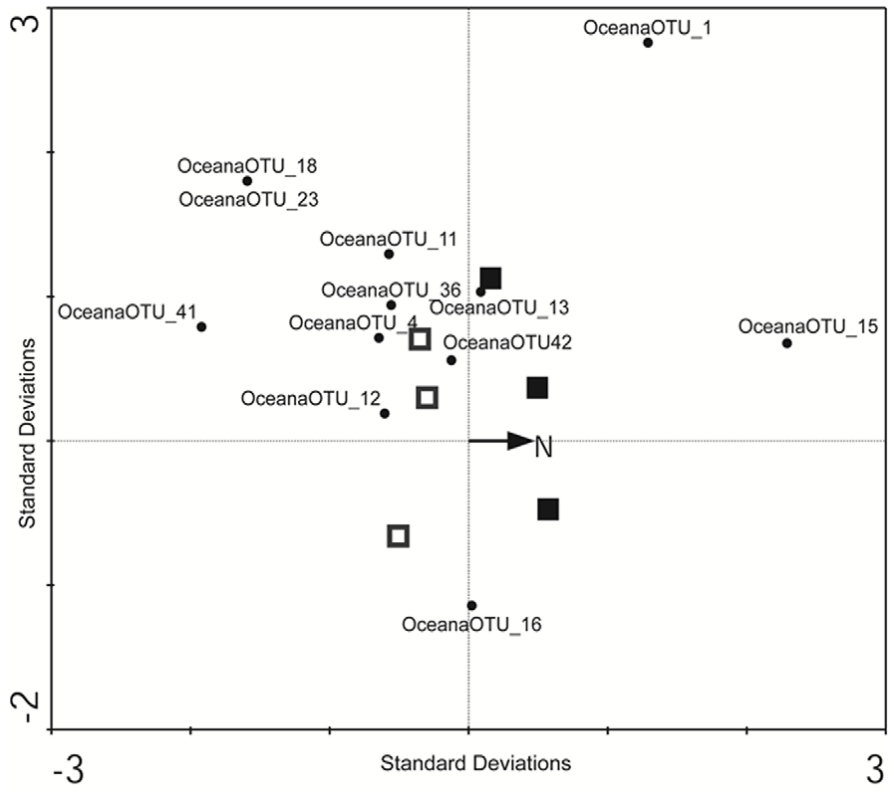
Triplot based on a Canonical Correspondence Analysis showing the relationships between samples of a fungal community developing under ambient conditions of N deposition (open squares) and under conditions of simulated elevated N depostion (closed squares), the relative abundance of the 12 most widespread fungal Operational Taxonomic Units (OTUs, black circles), and total N depostion (vector) in the forest floor of a maple dominated hardwood ecosystem. The primary axis accounts for 22% of the variance in OTU relative abundances; the second axis a further 37%. The relationship between fungal relative abundances and total N is not significant (Monte Carlo P = 0.52). OTU codes correspond to the phylogenies (Fig. 2, Fig. 3): OceanaOTU_1, *Typhula* sp; OceanaOTU_4, *Mycena* sp; OceanaOTU_11, *Entoloma* sp; OceanaOTU_12, *Athelia* sp.; OceanaOTU_13, *Gomphales* sp.; OceanaOTU_15, *Ceratosebacina* sp.; OceanaOTU_16, *Ceratobasidium* sp.; OceanaOTU_18, *Sistotrema* sp.; OceanaOTU_23, unidentified Ascomycota; OceanaOTU_36, Leotiomycete sp.; OceanaOTU_41, Leotiomycete sp.; OceanaOTU_42, Leotiomycete sp.

We examined the effect of simulated N deposition on fungal community composition at the most southerly of our sites (site D, Table 1), in which consistently lower levels of fungal *cbh*I and *lcc* gene expression under simulated N deposition were observed in 2007 and 2009. We recovered a species-rich and diverse active fungal community from the forest floor of this site (Figs 2, 3). Although we recovered a higher diversity of ascomycete than basidiomycete sequences, basidiomycete sequences clearly dominated the rRNA gene library, and, moreover, represented 66% of the most widespread species at this site (Fig. 4). Lignin decomposition is principally associated with basidiomycete species, and *Clitocybe*, *Collybia*, *Marasmius*, and *Mycena* species are well known for their ligninolytic capability and are commonly recovered from forest floor environments [13], [30], [31]. We recovered taxa from *Mycena*, *Clitocybe*, *Crepidotus* and *Clitopilus* (Fig. 3), as well as *Entoloma* and *Typhula* that are also most likely saprotrophs, and these represented approximately half of the basidiomycete diversity. The most abundant and widespread basidiomycete taxa however, were members of the Cantharellales, Ceratobasidiales and Atheliales. The nutritional mode of these resupinate taxa is unclear; they are most likely saprotrophs, but mycorrhizal and parasitic life-strategies are also known from these groups [36]. A Gomphalian taxon recovered under both ambient and simulated N conditions is probably mycorrhizal, and Tremellomycete yeasts were also recovered under both treatments. Ascomycetes were predominantly recovered from the Dothideomycetes, Sordariomycetes and Leotiomycetes, although most remained unidentified at better than ordinal level (Fig. 2). This was especially true within the Leotiomycetes (Fig. 2). Although simulated N deposition had no significant effect on the relative abundance of the more widespread basidiomycete or ascomycete taxa, the proportion of ascomycete species in the active community was nevertheless significantly reduced under simulated N deposition. The consequences of the apparent reduction in ascomycete diversity during the later stages of litter decomposition are largely unknown; *lcc* is broadly distributed among the Basidiomycota, and less broadly among the Ascomycota and other fungi [35]. With the exception of some species of Sordariomycetes (e.g., *Xylaria*), saprotrophic ascomycetes isolated from forest soils appear to be primarily cellulolytic and chitinolytic organisms, rather than agents of lignin degradation [14], [15], [16]. Indeed, ascomycete-derived cellolulytic and chitinolytic genes have been recovered in a previous transcriptomic analysis of this ecosystem [37]. The reduced transcription of *lcc* that we observed may then be the result of basidiomycetes expressing less *lcc* as a physiological response to higher N availability; however, the extent of laccase distribution among ascomycetes is poorly understood [35]. Leotiomycete ecologies are generally considered to be plant-based, include pathogenic, endophytic, saprotrophic and mycorrhizal life strategies [38], and laccase positive species (e.g. *Botryotinia fukeliana*) are known [39]. Similarly, within the Dothideomycetes and Sordariomycetes, many of the taxa we recovered appear to place with plant pathogens such as *Mycosphaerella*, *Neonectria* and the *Cryphonectriaceae*. Species within these groups may use laccase as an “attack” enzyme during infection, and subsequently to break down senescent plant cells [39]. Although these taxa were sparsely distributed across the site, the possibility that their absence from the simulated N deposition treatment is in part responsible for the decline in *lcc* gene expression and laccase activity cannot be discounted. Although ligninolytic basidiomycetes are often considered the primary agents of late-stage litter decomposition [30], [35], [40], our results emphasize the need for further studies to connect functional genes recovered in the transcriptome to the species active in the community.

### Conclusions

Despite their important function in plant litter decay and nutrient recycling [41], [42], relatively little is known about the sensitivity of saprotrophic fungal communities and community function to environmental change [18], [43]. We examined relative levels of functional gene transcription and community composition in a northern hardwood ecosystem in which chronic elevated N deposition has resulted in the slowing of litter decay and greater soil C sequestration. Our results indicate that the transcription of a key oxido-reductive gene (*lcc*) involved in lignin decomposition is lower under elevated N deposition, suggesting that the physiological response of saprotrophic fungi to higher N availability may be an important link between environmental change and ecosystem function. At the same time, our results indicate that while the relative abundances of the more common fungi were unaffected by elevated N deposition, fewer ascomycete species were recovered in the latter stages of decomposition. Our results emphasize the ongoing need to clarify the functional potential and ecological niche of fungal species, as well as the need to understand how that potential is realized under varying environmental conditions. Overall, our results indicate that chronic N deposition can elicit both compositional and functional changes in fungal communities. These changes may mechanistically underlie the slowing of decay and increased soil C storage associated with N deposition in this ecosystem.

## Materials and Methods

### Study sites and soil sampling

The influence of chronic atmospheric N deposition was investigated in four sugar maple (*Acer saccharum* Marsh.) dominated stands distributed across lower and upper Michigan (Table. 1). Overstory associates include *Quercus rubra* L., *Fraxinus americana* L., *Betula alleghaniensis* Britt. and *Prunus serotina* Ehrh.. The forest floor is composed of a thin Oi horizon dominated by relatively intact sugar maple leaf litter, and a thicker Oe horizon interpenetrated by a dense root mat. Mineral soils are sandy (85–90% sand) typic Haplothods of the Kalkaska series. All four sites are floristically and edaphically similar, but differ in mean annual temperature, ambient atmospheric N deposition, and soil N availability [7], [20]. At each site, three 30-m x 30-m plots receive ambient atmospheric N deposition and three 30-m x 30-m plots receive simulated atmospheric N deposition. The simulated atmospheric N deposition treatment (30 kg N ha^−1^ y^−1^) was initiated in 1994 and consists of 6 equal applications of NaNO_3_ delivered as dry pellets to the forest floor over the growing season; NO_3_^−^ composes ∼60% of wet plus dry atmospheric N deposition in this region. Soil sampling was performed in November 2007 (site D only) and October 2009 (all sites) after leaf senescence. In each of the six plots at each site, 10 random 0.1-m×0.1-m forest floor samples (Oe horizon) were collected, composited, and homogenized in order to ensure plot coverage and representation of all overstory tree species. Parts of the homogenized samples were immediately flash-frozen in liquid N_2_ to prevent RNA degradation, and the remainder, subject to enzyme measurements, was transported on ice and later stored at -20 °C.

### RNA extraction and cDNA preparation

In both 2007 (at Site D) and 2009 (at all four sites), total RNA was extracted using a previously published protocol [44]. Briefly, the RNA from ∼1 g of forest floor was extracted using glass beads and a phenol-based solution. The samples were disrupted using the FastPrep FP120A instrument (MP Biomedicals, Solon, USA) for 30 s at a speed of 6.5. The RNA of this crude extract was then centrifuged, precipitated with ethanol, and separated using RNA/DNA Midi kits (Qiagen, Hilden, Germany) as recommended by the manufacturer. Before further purification of the RNA using the RNeasy Plant Mini kit (Qiagen), a DNAse step (Qiagen) was added as recommended by the manufacturer. To create cDNA, 3 µl of the purified DNA-free RNA was used as template in an adaptor, polydT primed reverse transcriptase-PCR, and further processed to synthesize the cDNA via 17 to 21 cycles of a long-range PCR using the SMART™ PCR cDNA Synthesis & Advantage 2 PCR Kits (Clontech, Mountain View, USA). One cDNA was created for each of the experimental plots.

### Cellobiohydrolase and laccase gene relative expression

We initially screened all cDNA for cellobiohydrolase (*cbhI*), laccase (*lcc*), and β-tubulin gene sequences using previously described primer sets and PCR conditions [33], [44], [45], [46]. Selectivity of the primers for fungal functional genes was confirmed by cloning and sequencing and subsequent BlastP searches of the corresponding amino acid sequence. Based on sequenced expressed *lcc* genes, the *lcc* primer pair was modified and adjusted to reduce the degree of degeneracy from 32- and 64-fold to 8-fold. Hence, in the quantitative PCR (qPCR) approach, the new primer pair Cu1Fmod1 (5′- ACG GTY CAY TGG CAY GG -3′) and Cu2Rmod1 (5′- GRC TGT GGT ACC AGA AIG TNC -3′) was used. However, the both original *lcc* primer pairs were also used and provided the same relative results, but with a slightly lower sensitivity.

Quantitative PCR (qPCR) was performed using a Mx3000P (Stratagene) real-time PCR system and Brilliant SYBR Green qPCR Master Mix (Stratagene). QPCR-cycling parameters were a 10 min denaturation at 95 °C followed by 40 cycles of 30 s at 94 °C, 1 min at 50 °C, and 2 min at 72 °C with fluorescence measurement during the extension step. A melting curve was obtained by heating to 95 °C for 1 min, cooling to 55 °C for 30 s, and then ramping up the temperature to 95 °C at 0.5 °C min^−1^. Fluorescence data were collected continuously during the ramp. Each cDNA was serial-diluted 10, 20, 40, 80 and 160-fold, and triplicate measurements of all genes made at each dilution in order to confirm that relative threshold amplification values (ΔC_T_) were independent of starting cDNA template concentration [47].

To test the hypothesis that simulated N deposition suppresses ligno-cellulolytic gene expression, we calculated 2^−ΔΔC^_T_ for *cbh*I and *lcc* in each cDNA with β-tubulin as the housekeeping gene [47]. Because both the rRNA gene library and the functional gene melting curves showed that the cDNA represented complex gene mixtures, the use of single species – single gene calibrators was inappropriate; we therefore calculated the mean C_T_ value for each gene within the ambient N deposition plots at each site, and then used these as the standard from which to calculate 2^−ΔΔC^_T_ for each sample at that site. Samples were ranked by their relative expression levels, and the significance of the difference between ambient N and simulated N deposition was determined using the non-parametric Wilcoxon-Mann U test.

### Extracellular Enzyme Assays

Potential cellobiohydrolase and phenol-oxidase enzyme activities were determined at Site D in 2007, and at all four sites in 2009. Extracellular enzymes were extracted from 1 g of litter suspended in 125 mL 50 mM acetate buffer (pH 5) using a Bio Homogenizer M133 (Biospec, Bartlesville, USA). The suspension was continuously stirred and 50 µL aliquots were dispensed in 96-well microplates, with 8–16 analytical replicates per sample. Laccase activity was measured spectrophotometrically via 3 min interval-kinetic in a EL800 platereader (Bio-Tek Instruments, Winooski, USA) using 3 mM ABTS (ε_470_  = 27.5 mM^−1^ cm^−1^) as the substrate (Sigma, St. Louis, USA). Cellobiohydrolase activity was determined using a fluorometric assay, in which methylumbelliferone-linked cellobiose was used as the substrate [48]. Enzyme activity was expressed on a dry weight basis.

### Ribosomal rRNA Gene Library

In 2007, fungal 28S rRNA gene fragments (LSU, large subunit) were amplified from cDNA obtained from Site D using the primers LR0R and LR3. Primer sequences were obtained from http://www.biology.duke.edu/fungi/mycolab/primers.htm. PCR cocktails included 50 ng cDNA, 200 nM dNTPs, 1X 1.5 mM MgCl_2_ PCR buffer (Roche), 0.5 µM of each primer, and 50 µg of BSA. After an initial denaturation step of 3 min at 94°C, 20 cycles of 94°C for 30 s, 55°C for 45 s and 72°C for 90 s and a final extension step of 72°C for 15 min were carried out using Stratagene PCR cyclers (La Jolla, CA). PCR products were gel-purified using the QIAquick Gel Extraction Kit (Qiagen), and cloned into the pCR 2.1-TOPO vector using the TOPO TA Cloning kit (Invitrogen, Carlsbad, USA) according manufacturer protocol. Altogether, 48 clones from each of the 6 cDNA were randomly picked, cultured overnight in liquid Luria broth and bi-directionally sequenced at the Laboratory for Genomics and Bioinformatics at the University of Georgia using standard M13 primers. Contigs were constructed using Geneious 5.3 (BioMatters Ltd, Auckland, NZ).

### Definition of Operational Taxonomic Units and Phylogenetic Analysis

The rDNA 28S sequences were aligned using the FFT-NSi x1000 algorithm in MAFFT [49], and a bootstrapped neighbour-joining tree constructed using the Kimura 2-parameter model and pair-wise deletion of gaps in MEGA 4.0 [50]. Each phylogenetically unique sequence was considered as an operational taxonomic unit (OTU); moreover, clusters of sequences forming well-supported terminal groups (bootstrap >95) were considered members of an OTU, and subsequent alignments of these members revealed mean pairwise sequence similarities of 97.2 to 99.9%. A sequence representative of each OTU was BLAST searched against the NCBI nrDNA database, and the top five matches downloaded. Generally, we avoided unidentified environmental sequences, even if they were the top match, because of their limited utility in taxon identification. For phylogenetic analysis, Ascomycete and Basidiomycete sequences were considered separately. OTU and reference sequences were aligned using MAFFT, and the alignment edited to remove ambiguously aligned regions including the D1 and D2 variable domains. Maximum Parsimony was conducted in MEGA 4.0 using the Close-Neighbour-Interchange algorithm and a randomly generated starting tree; bootstrapping used 100 replications. OTU sequences were deposited at GenBank under accession numbers FJ040343 – FJ403395.

### Fungal Community Analysis

The relationship between simulated N deposition and the relative abundances of fungal OTU was assessed for the community active at site D in 2007 using a Canonical Correspondence Analysis (CCA; [51]). The relative abundance of each OTU within each of the six plots at Site D was estimated as a percentage of the total number of sequences in the ribosomal rRNA library. Nitrogen availability in the forest floor of each plot was estimated as the annual sum of simulated and ambient N deposition (g ha^−1^ 2007). For CCA, we restricted the analysis to the 12 OTU that were recovered from at least two of the six plots at Site D, and tested the strength of the correlation between nitrogen availability and fungal community similarity through Monte Carlo simulation with 500 replications in Canoco Vs. 4.54 (Biometris, Wageningen, NL). Because variation in soil moisture content can influence the distribution of fungal species [16], the mean gravimetric water content (% mass after 48 hrs at 105°C) of the forest floor in each of the six plots was included as a covariable in the analysis.

## References

[1] Berg B, Matzner E (1997). Effect of N deposition on decomposition of plant litter and soil organic matter in forest systems.. Environ Rev.

[2] Galloway JN, Dentener FJ, Capone DG, Boyer EW, Howarth RW (2004). Nitrogen cycles; past, present and future.. Biogeochem.

[3] Nadelhoffer KJ, Emmet BA, Gundersen P, Kjønnas OJ, Koopmans CJ (1999). Nitrogen deposition makes a minor contribution to carbon sequestration in temperate forests.. Nature.

[4] De Vries W, Reinds GJ, Gundersen P, Sterba H (2006). The impact of nitrogen deposition on carbon sequestration in European forests and forest soils.. Global Change Biol.

[5] Magnani F, Mencuccini M, Borghetti M (2007). The human footprint in the carbon cycle of temperate and boreal forests.. Nature.

[6] Waldrop MP, Zak DR, Sinsabaugh RL, Gallo M, Lauber C (2004). Nitrogen deposition modifies soil carbon storage through changes in microbial enzymatic activity.. Ecol Appl.

[7] Zak DR, Holmes WE, Burton AJ, Pregitzer KS, Talhelm AF (2008). Simulated atmospheric NO_3_
^-^ deposition increases soil organic matter by slowing decomposition.. Ecol Appl.

[8] Pregitzer KS, Burton AJ, Zak DR, Talhelm AF (2008). Simulated chronic nitrogen deposition increases carbon storage in Northern Temperate forests.. Global Change Biol.

[9] DeForest J, Zak DR, Pregitzer KS, Burton AJ (2004). Atmospheric nitrate deposition, microbial community composition, and enzyme activity in northern hardwood forests.. Soil Sci Soc Am J.

[10] Burton AJ, Pregitzer KS, Crawford JN, Zogg GP, Zak DR (2004). Chronic NO_3_
^-^ additions reduce soil respiration in northern hardwood forests.. Global Change Biol.

[11] Carreiro MM, Sinsabaugh RL, Repert DA, Parkhurst DF (2000). Microbial enzyme shifts explain litter decay responses to simulated nitrogen deposition.. Ecology.

[12] Kirk KT, Farrell RL (1987). Enzymatic “combustion”: the microbial degradation of lignin.. Ann Rev Microbiol.

[13] Osono T (2007). Ecology of ligninolytic fungi associated with leaf litter decomposition.. Ecol Res.

[14] Osono T, Takeda H (2002). Comparison of litter decomposing ability among diverse fungi in a cool temperate deciduous forest in Japan.. Mycologia.

[15] Worrall JJ, Anagnost SE, Zabel RA (1997). Comparison of wood decay among diverse lignicolous fungi.. Mycologia.

[16] Baldrian P, Merhautová V, Petránková M, Cajthaml T, Šnajdr J (2010). Distribution of microbial biomass and activity of extracellular enzymes in a hardwood forest soil reflect soil moisture content.. Appl Soil Ecol.

[17] Sinsabaugh RL, Carreiro MM, Repert DA (2002). Allocation of extracellular enzymatic activity in relation to litter composition, N deposition, and mass loss.. Biogeochem.

[18] Allison SD, Hanson CA, Treseder KK (2007). Nitrogen fertilization reduces diversity and alters community structure of active fungi in boreal ecosystems.. Soil Biol Biochem.

[19] Fogg K (1988). The effect of added nitrogen on the rate of decomposition of organic matter.. Biol Revs Camb Phil Soc.

[20] Hassett JE, Zal DR, Blackwood CB, Pregitzer KS (2009). Are basidiomycete laccase gene abundance and composition related to reduced ligninolytic activity under elevated atmospheric NO_3_
^-^ deposition in a Northern hardwood forest?. Microb Ecol.

[21] Aro N, Pakula T, Penttilä M (2005). Transcriptional regulation of plant cell wall degradation by filamentous fungi.. FEMS Microbiol Rev.

[22] Lockington RA, Rodbourn L, Barnett S, Carter CJ, Kelly JM (2002). Regulation by carbon and nitrogen sources of a family of cellulases in *Aspergillus nidulans*.. Fung Gens Biol.

[23] Baldrian P, Valášková V (2008). Degradation of cellulose by basidiomycetous fungi.. FEMS Microbiol Rev.

[24] Ilmén M, Saloheima A, Onnela M-L, Penttilä ME (1997). Regulation of cellulase gene expression in the filamentous fungus *Trichoderma reesei*.. Appl Environ Microbiol.

[25] Jeffries TW, Choi S, Kirk TK (1981). Nutritional regulation of lignin degradation by *Phanerochaete chrysosporium*.. Appl Environ Microbiol.

[26] Leatham GF, Kirk TK (1983). Regulation of ligninolytic activity by nutrient nitrogen in white-rot basidiomycetes.. FEMS Microbiol Let.

[27] Li D, Alic M, Gold MH (1994). Nitrogen regulation of lignin peroxidase gene transcription.. Appl Environl Microbiol.

[28] Soden DM, Dobson ADW (2001). Differential regulation of laccase gene expression in *Pleurotus sajor-caju*.. Microbiology.

[29] Chen DM, Bastias BA, Taylor AFS, Cairney JWG (2003). Identification of laccase-like genes in ectomycorrhizal basidiomycetes and transcriptional regulation by nitrogen in *Piloderma byssinum*.. New Phytol.

[30] Frankland J (1998). Fungal succession – unravelling the unpredicatable.. Mycol Res.

[31] Osono T, Takeda H (2001). Effects of organic chemical quality and mineral nitrogen addition on lignin and holocellulose decomposition of beech leaf litter by *Xylaria* sp.. Euro J Soil Biol.

[32] Pointing SB, Pelling AL, Smith GJD, Hyde KD (2005). Screening of basidiomycetes and xylariaceous fungi for lignin peroxidase and laccase gene-specific sequences.. Mycol Res.

[33] Edwards IP, Upchurch RA, Zak DR (2008). Isolation of fungal Cellobiohydrolase I genes from sporocarps and forest soils by PCR.. Appl Environ Microbiol.

[34] Kellner H, Luis P, Zimdars B, Kiesel B, Buscot F (2008). Diversity of bacterial laccase-like multicopper oxidase genes in forest and grassland Cambisol soil samples.. Soil Biol Biochem.

[35] Baldrian P (2006). Fungal laccases – occurrence and properties.. FEMS Microbiol Rev.

[36] Binder M, Hibbett DS, Larsson KH, Larsson E, Langer E (2005). The phylogenetic distribution of resupinate forms across the major clades of mushroom-forming fungi.. System Biodiver.

[37] Kellner H, Zak Dr, Vandenbol M (2010). Fungi unearthed: Transcripts encoding lignocellulolytic and chitinolytic enzymes in forest soil.. PLoS ONE.

[38] Spatafora JW, Johnson D, Sung GH, Hesse C, O'Rourke B (2006). A five-gene phylogenetic analysis of the Pezizomycotina.. Mycologia.

[39] Lyons JL, Newell SY, Buchan A, Moran MA (2003). Diversity of ascomycete laccase gene sequences in a southeastern US salt marsh.. Microb Ecol.

[40] Baldrian P, Vořišková J, Dobiášová P, Merhautová V, Lisá L (2010). Production of extracellular enzymes and degradation of biopolymers by saprotrophic microfungi from the upper layers of forest soil.. Plant Soil.

[41] Rayner ADM, Boddy L (1988). *Fungal Decomposition of Wood, its Biology and Ecology*..

[42] Lindahl BD, Ihrmark K, Boberg J, Trumbore SE, Högberg (2006). spatial separation of litter decomposition and mycorrhizal nitrogen uptake in a boreal forest.. New Phytol.

[43] Edwards IP, Zak DR (2011). Fungal community composition and function after long-term exposure of northern forest to elevated atmospheric CO_2_ and tropospheric O_3_.. Global Change Biol.

[44] Luis P, Kellner H, Martin F, Buscot F (2005). A molecular method to evaluate basidiomycete laccase gene expression in forest soils.. Geoderma.

[45] Kellner H, Luis P, Buscot F (2007). Diversity of laccase-like multicopper oxidase (LMCO) genes in Morchellaceae: identification of genes potentially involved in extracellular activities related to plant litter decay.. FEMS Microbiol Ecol:.

[46] Einax E, Voight K (2003). Oligonucelotide primers for the universal amplification of β-tubulin genes facilitate phylogenetic analyses in the *regnum* Fungi.. Org Divers Evol.

[47] Livak KJ, Schmittgen TD (2001). Analysis of relative gene expression data using real-time quantitative PCR and the 2^-ΔΔC^
_T_ method.. Methods.

[48] Saiya-Cork KR, Sinsabaugh RL, Zak DR (2002). The effects of long-term Nitrogen deposition on extracellular enzyme activity in *Acer saccharum* forest soil.. Soil Biol Biochem.

[49] Katho K, Misawa K, Kuma KI, Miyata T (2002). MAFFT: a novel method for rapid multiple sequence alignment based on fast Fourier transform.. Nucl Acids Res.

[50] Tamura K, Dudley J, Nei M, Kumar S (2007). MEGA4: Molecular Evolutionary Genetics Analysis (MEGA) software version 4.0.. Mol Biol Evol.

[51] Ter Braak CJF (1986). Canonical correspondence analysis: a new eigenvector method for multivariate direct gradient analysis.. Ecology.

